# Differential gene and lncRNA expression in the lower thoracic spinal cord following ischemia/reperfusion-induced acute kidney injury in rats

**DOI:** 10.18632/oncotarget.18584

**Published:** 2017-06-20

**Authors:** Qing-Quan Liu, Hui Liu, Zhi-Gang He, Shi-Jie Zhang, Bao-Wen Liu, Le Wang, Wen-Hui Qiu, Qing Xu, Hong-Bing Xiang, Yong-Man Lv

**Affiliations:** ^1^ Department of Nephrology, Tongji Hospital of Tongji Medical College, Huazhong University of Science & Technology, Wuhan, Hubei, PR China; ^2^ Department of Anesthesiology and Pain Medicine, Tongji Hospital of Tongji Medical College, Huazhong University of Science & Technology, Wuhan, Hubei, PR China

**Keywords:** renal ischemia/reperfusion, lncRNA, spinal cord, high-throughput sequencing, transcriptomes

## Abstract

We used high-throughput RNA sequencing to analyze differential gene and lncRNA expression patterns in the lower thoracic spinal cord during ischemia/reperfusion (I/R)-induced acute kidney injury (AKI) in rats. We observed that of 32662 mRNAs, 4296 out were differentially expressed in the T8-12 segments of the spinal cord upon I/R-induced AKI. Among these, 62 were upregulated and 34 were downregulated in response to I/R (FDR < 0.05, |log_2_FC| > 1). Further, 52 differentially expressed lncRNAs (35 upregulated and 17 downregulated) were identified among 3849 lncRNA transcripts. The differentially expressed mRNAs were annotated as “biological process,” “cellular components” and “molecular functions” through gene ontology enrichment analysis. KEGG pathway enrichment analysis showed that cell cycle and renin-angiotensin pathways were upregulated in response to I/R, while protein digestion and absorption, hedgehog, neurotrophin, MAPK, and PI3K-Akt signaling were downregulated. The RNA-seq data was validated by qRT-PCR and western blot analyses of select mRNAs and lncRNAs. We observed that Bax, Caspase-3 and phospho-AKT were upregulated and Bcl-2 was downregulated in the spinal cord in response to renal injury. We also found negative correlations between three lncRNAs (TCONS_00042175, TCONS_00058568 and TCONS_00047728) and the degree of renal injury. These findings provide evidence for differential expression of lncRNAs and mRNAs in the lower thoracic spinal cord following I/R-induced AKI in rats and suggest potential clinical applicability.

## INTRODUCTION

Acute kidney injury (AKI) is a major renal disease with increasing incidence and mortality [1]. Clinically, AKI occurs due to renal or extra-renal surgery-induced ischemia/reperfusion (I/R), sepsis, and nephrotoxicity [2, 3]. The current primary therapeutic strategies for AKI, which includes renal replacement, symptomatic relief and supportive treatment, still result in high mortality rates of 40-80% [4, 5]. Some AKI patients cannot be treated effectively due to complications. Therefore, there is an urgent need to develop new treatment strategies that can significantly improve outcomes.

The efferent and afferent sympathetic nerves play a crucial physiological role in regulating renal function [6–13]. Activation of renal sympathetic nervous system has been implicated in ischemic AKI [14, 15]. Larkin *et al* reported that spinal T9 stimulation diminished renal blood flow and resulted in acute renal failure [16]. Our previous study showed that the T9 spinal cord segment was primarily involved in sympathetic regulation of renal function [17]. In addition, the excitability of renal sympathetic nervous system increased during I/R-induced acute renal failure [18, 19]. Therefore, blocking of renal sympathetic nervous activation or denervated kidneys before renal ischemia ameliorated post-ischemic renal injury to some extent [20–22]. Previous studies also demonstrated that neuromodulation therapy benefitted heart failure patients in preclinical and small-sized clinical studies [23, 24]. Hence, we postulated that spinal cord played an important role in acute kidney injury.

In recent years, significant progress has been made in understanding the function of renal sympathetic nervous system in the pathophysiology of AKI. However, the details regarding spinal cord involvement in AKI are unknown. Hence, we used RNA-seq with a higher sequencing depth as a systems biology approach in a rat model of I/R-induced AKI to identify the full transcriptome of the lower thoracic segments of spinal cord. This novel molecular biological technique allows identification of new protein-coding transcripts and novel non-coding RNA transcripts that play critical roles in many biological processes [25–28]. Since full transcriptome analysis of spinal cord from I/R-induced AKI is not available, we performed whole transcriptome sequencing and subsequent bioinformatic analysis to identify changes in mRNA and lncRNA expression in the lower thoracic segments of spinal cord in response to renal injury.

## RESULTS

### Validation of I/R-induced acute kidney injury in rats

**Figure 1:**
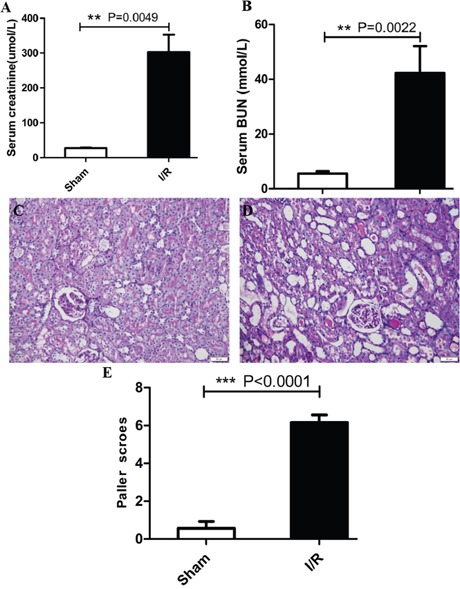
Renal function and renal histopathologic changes of I/R-induced AKI **(A)** Serum creatinine, **(B)** serum BUN (n=6, per group). **(C and D)** Representative images of kidney sections from sham group and I/R-induced AKI group. **(E)** Semiquantitative scoring of renal pathology injury. Results were expressed as mean ± SEM, **P <0.001, *** P<0.0001. Magnification, ×200, Bar, 50μm.

The functional analysis and histopathological validation was performed to ensure that all I/R-induced AKI rats used for sequencing experiments showed significant acute renal failure. We observed that 45 min of ischemia resulted in increased serum creatinine (302.5 ± 49.8μM in I/R; 27.2 ± 1.6μM in sham; P<0.01; Figure 1A), serum BUN (42.2 ± 9.9mM in I/R; 5.6 ± 0.9mM in sham; P<0.01; Figure 1B). Also, the I/R rats showed damaged tubular cells in the PAS stained tissue sections and high histological injury scores compared to sham rats (Figure 1C-1E). These results verified I/R-induced AKI in the rats used in our study.

### High throughput RNA-seq and genome-wide read mapping

High throughput RNA-seq was used to identify novel molecular players in the spinal cord that regulate renal function in the rat I/R-induced AKI model. Lllumina TrueSeq libraries were generated from total RNA isolated from the T8-12 of spinal cord tissues from 3 I/R and sham group rats, respectively. The flow chart of the sequencing strategy and analysis is shown in Supplementary Figure 1. To ensure accuracy of the subsequent bioinformatic analysis, low-quality reads and rRNA sequences were filtered prior to RNA-seq. We obtained over 85M of high quality sequence reads in each sample, which were mapped using Refseq (https://www.ncbi.nlm.nih.gov/refseq/). The mapped reads were assembled into putative transcripts by the TopHat software analysis (http://tophat.cbcb.umd.edu/) (Supplementary Table 1). The quality of overall transcriptome including saturation, duplication and coverage was assessed as shown in Supplementary Figure 2. Nearly 84.73%-91.47% of the reads mapped to the rat genome; about 79.43%-85.61% of the reads mapped to unique genomic regions among the aligned fragments, which were further verified for reliability. After aligning the sequences and identifying spliced junctions, we obtained 71906 novel transcripts.

### The expression profiling of mRNAs in spinal cord after I/R-induced AKI

**Figure 2:**
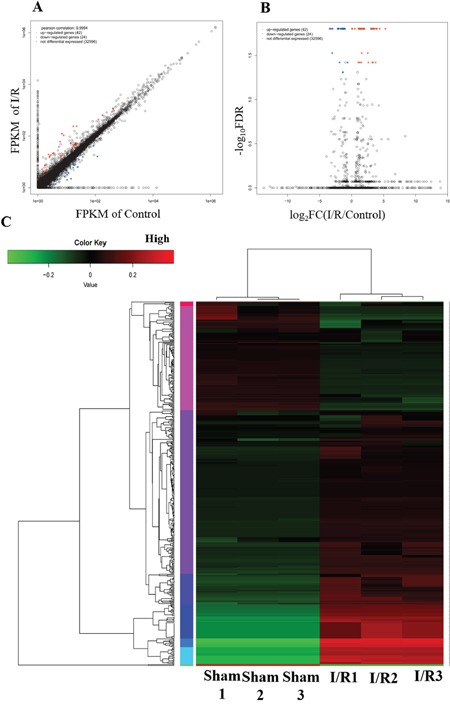
Differential expression genes in spinal cord from the I/R model of AKI **(A)** Scatter plot and **(B)** volcano plot for comparing global mRNA genes expression profiles in the spinal cord between the I/R and sham rat. Red color is indicative of up-regulated and blue color of down-regulated genes. Black color of is indicative of not statistical significant different genes when it do not pass the cutoff values of 1 and −1 in log_2_ scale and FDR (correctted p-value) <0.05. **(C)** Heat map showing hierarchical clustering of mRNA whose expression changes are more than twofold. In clustering analysis, up-and down-regulated genes are colored in red and green, respectively.

The mRNA expression was quantified using RPKM value (reads per kilobase per million mapped reads) to identify the gene expression changes in T8-12 spinal cord upon I/R induced AKI. Among the 32662 mRNAs that were measured by the RNA-seq, 2772 mRNAs were found deregulated upon I/R induced AKI by 2-fold, of which 1524 mRNAs were upregulated and 1248 mRNAs downregulated. Further analysis demonstrated 62 upregulated and 34 downregulated mRNAs in I/R (FDR<0.05, |log2FC|>1). The scatter and volcano plots analyses displaying the expression signatures of mRNAs are shown in Figure 2. The top 10 up-regulated and down-regulated mRNAs are listed in Tables 1 and 2.

**Table 1:** The detail information of the top 10 upregulation mRNAs in the T8-12 spinal cord after I/R-induced AKI

Gene name or ID	Description	Log2 FC (I/R/Sham)	P-value
Scarna15	small Cajal body-specific RNA 15	6.79	5.00E-05
ENSRNOG00000052828(novel)	defensin alpha 7	5.28	5.00E-05
Defa7	defensin alpha 7	4.37	5.00E-05
Defa5	defensin, alpha 5	3.93	5.00E-05
Prg2	proteoglycan 2	3.73	0.00015
RatNP-3b	neutrophil antibiotic peptide NP-3 precursor	3.57	5.00E-05
S100a9	S100 calcium binding protein A9	3.46	5.00E-05
ENSRNOG00000055892(novel)		3.35	0.00015
Epx	eosinophil peroxidase	3.34	0.00015
P2×7R	P2X purinoceptor 7 receptor	3.25	5.00E-05

**Table 2:** The detail information of the top 10 downregulation mRNAs in the T8-12 spinal cord after I/R-induced AKI

Gene name or ID	Description	Log2 FC (I/R/Sham)	P-value
ENSRNOG00000030339 (novel)		−12.70	1.00E-04
Smoc2	SPARC related modular calcium binding 2	−3.44	5.00E-05
Slc47a1	multidrug and toxin extrusion protein 1	−3.23	5.00E-05
Slc26a7	solute carrier family 26 (anion exchanger), member 7	−3.04	1.00E-04
Igfbp5	insulin-like growth factor binding protein 5	−2.97	5.00E-05
Dpp4	dipeptidylpeptidase 4	−2.06	5.00E-05
Col8a1	collagen, type VIII, alpha 1	−1.99	5.00E-05
Slc9a2	solute carrier family 9, subfamily A, member 2	−1.88	0.00015
Scara3	scavenger receptor class A, member 3	−1.7	5.00E-05
Vom2r44	vomeronasal 2 receptor 44	−1.52	5.00E-05

### The expression profiling of lncRNA in spinal cord after I/R-induced AKI

**Figure 3:**
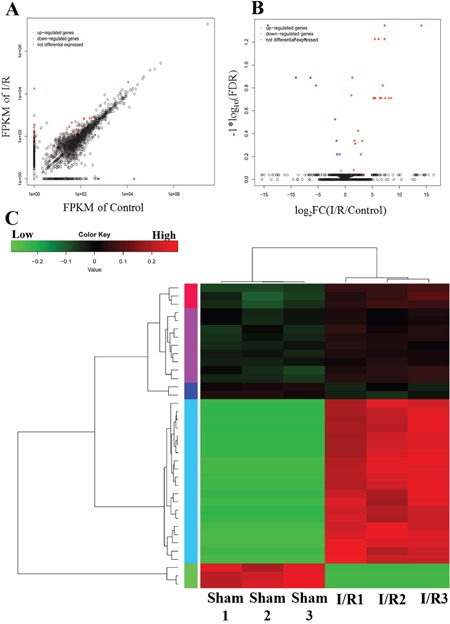
Differential expression lncRNA in the T8-T12 pinal cord from the I/R model of AKI **(A)** Scatter plot and **(B)** volcano plot for comparing global lncRNA expression profiles in spinal cord between the I/R and sham rat. Red color is indicative of up-regulated and blue color of down-regulated genes. Black color of is indicative of not statistical significant difference when it do not pass the cutoff values of 1 and −1 in log2 scale and p<0.05. **(C)** Heat map showing hierarchical clustering of lncRNA whose expression changes were more than twofold. In clustering analysis, up-and down-regulated genes are colored in red and green, respectively.

To explore the potential role of lncRNAs in the T8-12 spinal cord at 24 hours after I/R-induced AKI, the expression profiles of lncRNAs were determined by high throughput RNA-seq. Our analyses revealed 3849 lncRNA transcripts in the spinal cord from I/R and control groups, among which 2253 known and 1596 novel lncRNAs were identified by filtering the transcripts with CPC (Coding Potential Calculator), CNCI (Coding-Non-Coding Index) and Pfam analyses (Supplementary Figure 3). Among these, 35 upregulated and 17 downregulated lncRNAs were identified in the spinal cord responding to I/R-induced AKI (fold change >2, P < 0.05). Figure 3 show the expression signatures of lncRNAs by using scatter and volcano plots. The top 10 upregulated and downregulated lncRNAs were listed in Table 3.

**Table 3:** The detail information of the top 10 upregulation and 10 downregulation LncRNAs in the T8-12 spinal cord after I/R-induced AKI

Upregulation			Downregulation		
Transcript_id	Log_2_ FC (I/R/Sham)	P-value	Transcript_id	Log_2_ FC (I/R/Sham)	P-value
TCONS_00034035	14.09	0.0001	TCONS_00058568	−14.27	0.00325
TCONS_00047107	8.49	0.00325	TCONS_00047728	−9.23	0.00035
TCONS_00018621	8.02	0.0005	TCONS_00070166	−9.1	0.00105
TCONS_00033710	7.36	0.00325	TCONS_00033040	−7.41	0.00035
TCONS_00034216	7.269	0.0001	TCONS_00042175	−6.39	0.00105
TCONS_00004522	7.267	0.0003	TCONS_00015342	−5.42	0.00135
TCONS_00033384	6.95	0.00145	TCONS_00014262	−1.85	0.0055
TCONS_00055158	6.69	0.00325	TCONS_00070113	−1.59	0.0097
TCONS_00017521	6.53	0.00325	TCONS_00054344	−1.50	0.01425
TCONS_00010461	6.45	0.00345	TCONS_00050246	−1.01	0.0147

### Gene ontology annotation for differential expression genes

**Figure 4:**
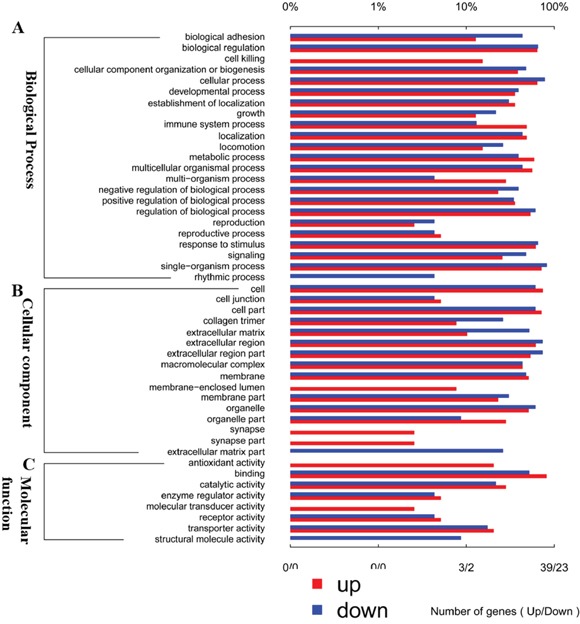
The differential expression of mRNA in spinal cord after I/R-induced AKI was analyzed by Gene Ontology (GO) annotation **(A)** Biological process classification, **(B)** cellular component classification and **(C)** molecular function classification. Red bar represents up-regulated genes, blue bar represents down-regulated genes.

The differentially expressed genes identified by the RNA-seq analyses were annotated using the GO database (Gene Ontology, http://www.geneontology.org/) into three biological functional groups, namely, biological process (Figure 4A), cellular component (Figure 4B) and molecular function (Figure 4C). We found that the differentially expressed mRNAs were primarily involved in the biological processes followed by cellular component GO functions.

### Gene ontology and KEGG Pathway enrichment analysis

**Figure 5:**
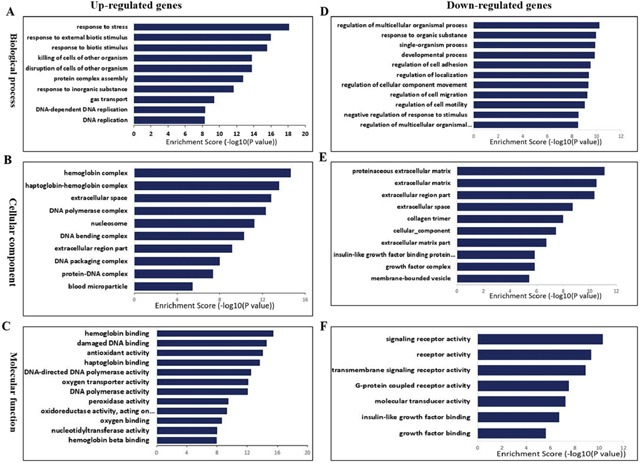
Biological functions of differentially expressed mRNAs The significant biological process **(A)**, cellular component **(B)**, and molecular function **(C)** of up-regulated mRNAs. The significant biological process **(D)**, cellular component **(E)** and molecular function **(F)** of down-regulated mRNAs.

We performed GO and KEGG (Kyoto Encyclopedia of Genes and Genomes) pathway enrichment analyses of deregulated genes to determine the molecular changes in the lower spinal cord tissue during I/R induced AKI. The top five enriched GO biological processes for upregulated genes in the I/R group included responses to stress, external biotic stimuli, external stimuli, killing of cells and disruption of cells (Figure 5A). The most enriched GO cellular components for up-regulated genes in I/R group included hemoglobin complex, haptoglobin-hemoglobin complex, extracellular space and DNA polymerase complex (Figure 5B). The enriched GO molecular functions for up-regulated genes in I/R group were hemoglobin binding, damaged DNA binding and antioxidant activity (Figure 5C).

The enriched GO biological processes for downregulated genes in I/R group were regulation of multicellular organismal process, response to organic substance, single-organism process and developmental process (Figure 5D). The enriched GO cellular components for down-regulated genes in I/R group included proteinaceous extracellular matrix, extracellular matrix, extracellular matrix part and extracellular space (Figure 5E). The enriched GO molecular function for down-regulated genes in I/R included signaling receptor activity (Figure 5F).

**Figure 6:**
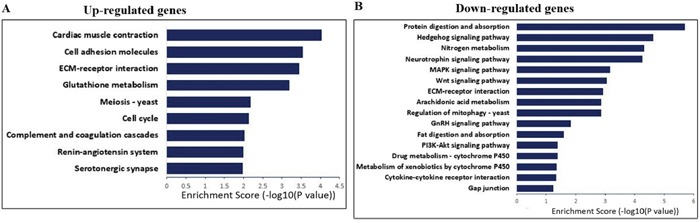
KEGG pathway analysis for up-regulated and down-regulated mRNAs **(A)** The significant pathways for up-regulated genes in lower spinal cord following I/R-induced AKI group. **(B)** The significant pathways for down-regulated genes in lower spinal cord following I/R-induced AKI group.

Next, we conducted KEGG pathway analysis for differentially expressed genes. The results showed that the up-regulated genes in I/R group were involved in cardiac muscle contraction, cell adhesion, cell cycle, renin-angiotensin system, and serotonergic synapse (Figure 6A). The downregulated genes in I/R group were involved in protein digestion and absorption, hedgehog signaling, neurotrophin signaling, MAPK signaling, and PI3K-Akt signaling pathways (Figure 6B).

### Real-time quantitative PCR and western blot validation

**Figure 7:**
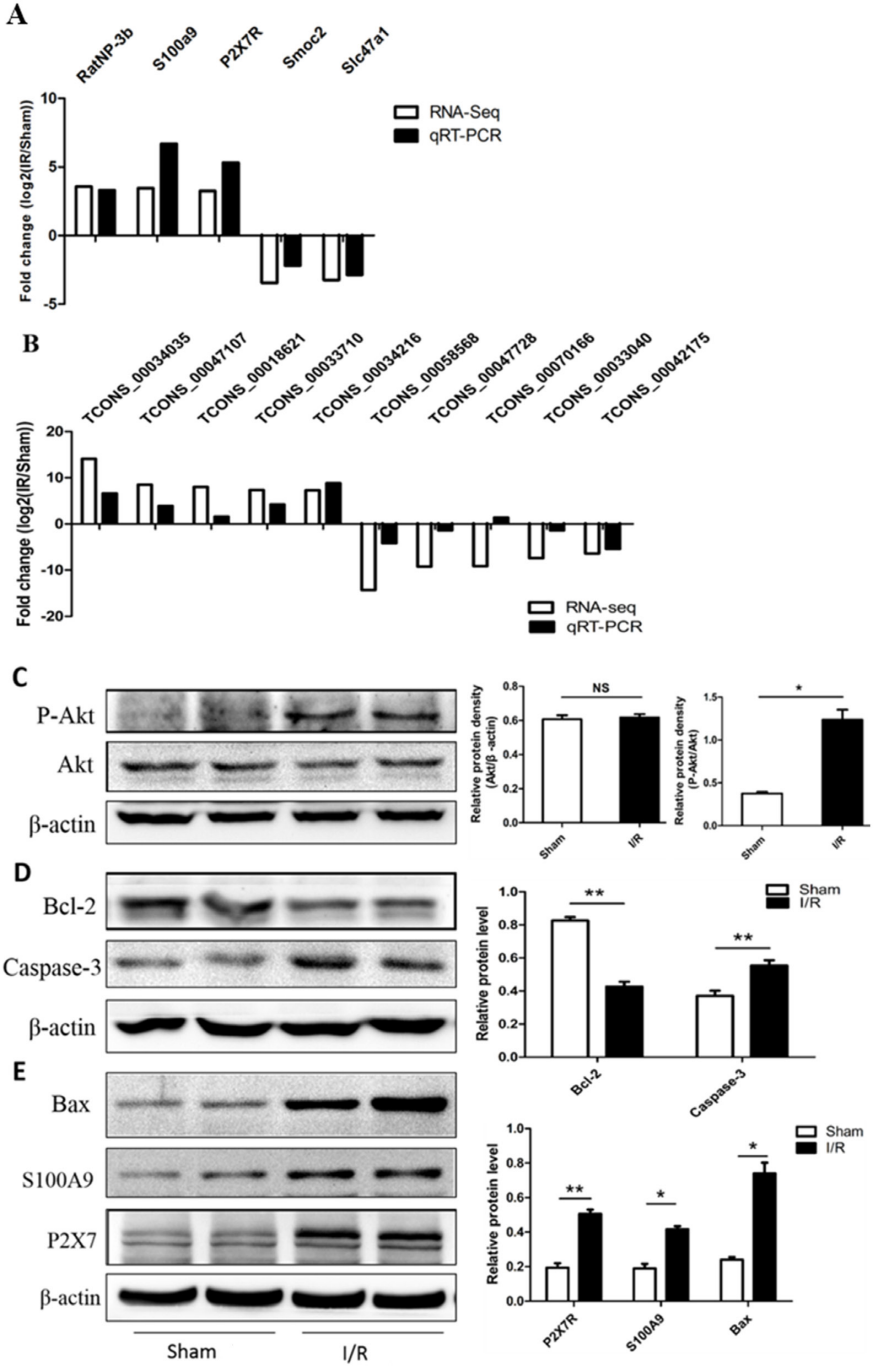
Validation of differential expression mRNAs and lncRNAs in spinal cord by real time RT-qPCR and Western blot analysis **(A)** The differential expression mRNA levels were validated by qRT-PCR. **(B)** Five upregulated lncRNAs and five downregulated lncRNAs were validated by qRT-PCR. The levels of mRNAs and lncRNAs were normalized to GAPDH and expressed as fold of change compared to sham group. The results represent the mean± SEM of three independent experiments. *p < 0.05; **p < 0.01 compared with the sham group. Western blot analysis shown protein expression levels of Akt, P-Akt **(C)**, Bcl-2, Caspase-3 **(D)**, P2×7R, S100A9, Bax **(E)**. Each bar represents the mean ± SEM for at least 6 animals. *p< 0.05, **p< 0.01 vs Sham group.

Then, we validated the high throughput RNA-seq results by performing qRT-PCR analysis of differentially expressed mRNAs and lncRNAs. Although the fold changes in mRNA and lncRNA were different between qRT-PCR and RNA-seq data, the expression patterns revealed similar conclusions (Figure 7A, 7B). Our RNA-seq results showed the genes of Bax, Caspase-3 and Akt were upregulated and Bcl-2 was downregulated in I/R group. So we using Western blot validated these differential expression genes at protein level, which showed that P2×7R, S100A9, Bax and P-Akt were up-regulated and Bcl-2 was downregulated in the lower spinal cord following I/R-induced AKI (Figure 7C-7E). These data indicated differential expression of the mRNAs and lncRNAs in the lower spinal cord tissue suggesting their involvement in the I/R-induced AKI. The qRT-PCR and western blot analyses were consistent with the high throughput RNA-seq data.

### Differential lncRNA expression is correlated with the degree of renal injury

**Figure 8:**
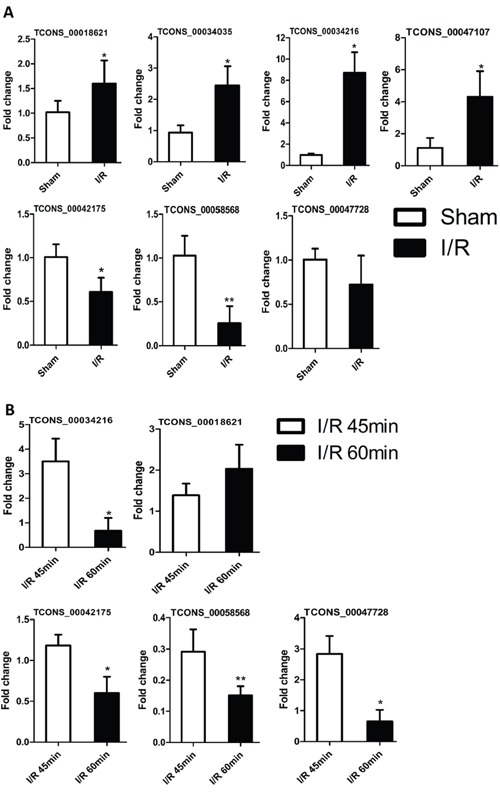
Changes in the expression levels of selected lncRNAs in renal pathological damage for AKI Real time RT-qPCR analyzed the expression profile of selected lncRNA. **(A)** Comparison between control group (Sham group) and I/R 45min. **(B)** Comparison between I/R 45min group and I/R 60min group. The lncRNA levels of each gene were normalized to GAPDH. The results represent the mean± SEM of three independent experiments. *p<0.05; **p<0.01.

As indicated previously, many lncRNA transcripts in the spinal cord were either upregulated or downregulated during I/R induced AKI. Compared with control group, we found lncRNA TCONS_00018621, lncRNA TCONS_00034035, lncRNA TCONS_00034216 and lncRNA TCONS_00047107 were significantly increased (P<0.05), and lncRNA TCONS_00042175 and lncRNA TCONS_00058568 were significantly decreased in AKI group (P<0.05), shown in Figure 8A. Further, whether these differential lncRNAs expression associated with degree of renal injury, we even built two different animal models, such as kidney ischemia for 45 mins (I/R 45min) and 60 mins (I/R 60min), which were considered two different degrees of pathological injury. The results showed that expression of lncRNA TCONS_00042175, TCONS_00058568, TCONS_00047728 and TCONS_00034216 was lower at 60 mins compared to 45 mins post-I/R (P<0.05 or P<0.001), and the expression of lncRNA TCONS_00018621 was higher in I/R 60min group than the I/R 45min group (Figure 8B). Interestingly, the expression of lncRNA TCONS_00034126 was 4-fold lower at 60 mins compared to at 45 mins (P<0.05, Figure 8B), however, compared with control group, the lncRNA TCONS_00034126 was obviously up-expressed in I/R group. Collectively, these results suggested that selected lncRNAs were either positively or negatively associated with the renal pathology injury induced by I/R.

## DISCUSSION

Next generation sequencing techniques are useful for detecting novel genes, transcriptional and epigenetic networks. Our study is the first to explore the whole transcriptome profiles of spinal cord in a I/R rat AKI model using next generation RNA sequencing and bioinformatics analysis. We identified many differentially expressed genes, pathways and biological processes in the spinal cord during renal injury. By high throughput RNA-seq, we identified 32662 mRNAs in the spinal cord tissue from both the I/R and sham groups, of which 62 upregulated and 34 downregulated mRNAs (FDR<0.05,|log2FC|>1) were identified in response to renal ischemia. Similarly, 3849 lncRNAs of which, 2253 were known and 1596 were novel were also identified by the RNA-seq analysis. Among these, 35 up-regulated and 17 down-regulated mRNAs were identified upon renal ischemia. From this list, we confirmed and verified few differentially expressed mRNAs and lncRNAs by qRT-PCR and western blotting analyses.

Previous studies have suggested that the lower thoracic segment of spinal cord controls renal function [13, 17, 29]. However, the exact segments of the spinal cord that are involved and the mechanisms operating in the spinal cord as a result of renal ischemia/reperfusion injury are unclear. Many studies have shown that lncRNAs play an important role in regulating gene expression [27, 30]. In this study, we found differential expression of many mRNAs and lncRNAs in the spinal cord further suggesting that the T8-12 spinal cord segment was involved in the neuronal response to renal injury. Although the functions of most lncRNAs are not fully known, our findings provide novel insights into their involvement in the molecular mechanism of renal failure.

Among the differentially expressed mRNAs, some including S100A9, RatNP-3b, SMOC2 and P2×7R have been reported earlier. Mitchell *et al*. reported that S100A8 and S100A9 expressing neutrophils traffic to the spinal cord during peripheral tissue inflammation, whereas, the S100A8 and S100A9 expression rapidly increased in response to carrageenan-induced inflammation of rat hind paws [31]. Sárvári *et al* demonstrated RatNP-3b up-regulation in estrogen receptor agonist-treated animals by Affymetrix Rat230 2.0 expression arrays and TaqMan-based quantitative real-time PCR [32]. Recently, Roy *et al*. analyzed the transcriptome in hypothalamus and cerebral cortex by RNA-seq analysis and found that SMOC2 expression was related to domestication [33]. Rudqvist *et al*. showed downregulation of Slc47a2 in the thyroid tissue by oligonucleotide microarray, 24h after I-131 administration in rats [34]. Previous studies showed enhanced expression of P2×7R in the spinal cord dorsal horn in a rat model of neuropathic pain induced by chronic constriction injury [35].

We further verified that 3 lncRNAs (TCONS_00042175, TCONS_00058568, TCONS_ 00047728) were negatively associated with the degree of renal ischemia injury (Figure 8), they further demonstrated involvement of development process of renal injury induced by I/R. Recently, lncRNAs were shown to be part of gene regulatory networks responding to nerve injury, neuropathic pain, renal fibrosis and acute rejection after renal allografts [36–39]. Further, lncRNA-ATB was identified as a novel biomarker of acute kidney rejection, which could identify patients with acute rejection and predict loss of kidney function [39]. Tu *et al*. reported that silencing lncNONRATT021972 decreased the P2×7R levels in the cervical sympathetic ganglia and improved cardiac function after myocardial ischemia [40, 41]. P2×7R expression was also strongly associated with kidney and nervous system diseases [42–45]. In this study, the P2X receptors P2×7R was upregulated in spinal cord after I/R-induced AKI group, consistent with previous studies that demonstrated increased P2X receptor expression in the stellate ganglia (SG) and superior cervical ganglia (SCG) after myocardial ischemia [46–48]. Moreover, several studies reported that the neuropeptide ghrelin or cholinergic agonists GTS-21 attenuated renal ischemia-reperfusion injury or sepsis-induced acute kidney injury through the vagus nerve [49–51]. Gigliotti *et al* and Inoue *et al* demonstrated that prior ultrasound prevented AKI by modulating the splenic neuroimmune axis, or stimulating the splenic cholinergic anti-inflammatory pathway, or vagus nerve stimulation [52–54]. Hence, we postulate that the list of novel lncRNAs (Table 3) found in our study represent new, yet undeciphered mechanisms in the response to renal injury.

Further, GO term enrichment analyses demonstrated that stress responses were the most enriched biological processes during I/R namely, response to external biotic stimuli and external stimuli. These results are consistent with previous studies showing activation of the afferent renal sympathetic nervous system during renal I/R [55]. The afferent renal nerves project to the ipsilateral dorsal horn in laminate I, III–V within the spinal cord [56] and play an important role in mediating renal ischemic injury-induced reduction in renal hemodynamic and renal functions. The most enriched GO molecular functions were DNA binding, antioxidant and signaling receptor activities. We found increased Bax and P-Akt in the spinal cord tissue following renal I/R. Conversely, we also observed decreased Bcl-2, but Akt expression was unaltered. These indicated that pro-apoptotic and PI3K/Akt signaling pathways were upregulated in the spinal cord in response to renal injury. Additionally, KEGG analysis showed upregulation of cell cycle and renin-angiotensin system genes, whereas, the downregulated genes during I/R included those involved in protein digestion and absorption, neurotrophin, MAPK and PI3K/Akt signaling pathways. These findings are consistent with previous studies that demonstrated activation renin-angiotensin system [57], nerve growth factor promoted growth arrest and apoptosis in tubular renal cells [58] after renal injury. The data collectively indicated that inhibition of apoptotic and PI3K/Akt signaling pathways in the spinal cord may represent potential therapeutic strategy to treat I/R induced renal injury.

In conclusion, we identified and validated differentially expressed mRNA and lncRNA transcripts in the lower thoracic spinal cord by high-throughput RNA-seq that maybe involved in I/R induced acute renal injury. Further detailed studies of factors uncovered by this analysis may serve as novel diagnostic markers and therapeutics for I/R induced AKI in the future.

## MATERIALS AND METHODS

### Animal care

All animal experiments were performed with adult male Sprague-Dawley (SD) rats (300 to 350g) in accordance with the guidelines according to the Huazhong University of Science and Technology Guide for the Care and Use of Laboratory Animals. All experimental animal procedures were approved by the Institutional Animal Care and Use Committee of Tongji Hospital. The rats were maintained and habituated in a standard 12h light-dark cycle with *ad libitum* access to food and drinking in a temperature-and humidity-controlled room (22°C ± 0.5°C, relative humidity 40%–60%).

### Acute kidney ischemia/reperfusion experiments

Rats were randomly divided into sham (control, n=12) and I/R (AKI, n=12) groups. They were anesthetized with 40mg/kg sodium pentobarbital intraperitoneal injection and placed on a heating pad to maintain a constant temperature. Acute kidney ischemia/reperfusion experiments were performed according to previously published protocols [59–62]. A midline abdominal incision was made and bilateral renal pedicles were exposed. Renal I/R were induced without trauma by clamping both renal pedicles for 45 minutes or 60 minutes. The ischemia was confirmed by visualizing dark color of kidneys. The blood flow was restored after clamp removal and the color of kidneys changed from dark to pink. To reduce abdominal air, 1ml warm normal saline was given intraperitoneally before abdominal closure. In sham controls, only bilateral renal pedicles were exposed. Rats were euthanized 24h after I/R, blood was drawn and their kidneys were thoroughly perfused with saline to remove any blood from the vascular beds. Kidneys and T8-12 spinal cord specimens were preserved at −80°C for further use.

### Analysis of renal function and histology

Serum creatinine (SCr), serum BUN levels were determined in the Automated Blood or Urine Chemical Analyzer Vitro 350 (Orthoclinical Diagnostic Inc., Rochester, NY). For histology, kidney specimens were first fixed with 10% formalin solution and embedded with paraffin. Then, 3μm thick renal tissue sections were cut and stained with periodic acid-Schiff reagent (PAS). Histological analysis was performed by experienced pathologists blinded to the experimental groups. The kidney injuries were graded according to the semi-quantitative scores developed by Paller *et al* [63, 64] as follows: (1) tubular epithelial smoothness or tubular expansion: score 1, (2) loss of brush-like edge: score 1or 2, (3) obstruction of tubular lumen: score 1 or 2, (4) cytoplasmic vacuolization: score 1, and (5) cell necrosis: score 1. The highest achievable score was seven.

### RNA isolation from spinal cord tissue

Total RNA from T8-12 segments of the spinal cord tissue from three rats was extracted for high-throughput sequencing using TRIzol® (Invitrogen, Carlsbad, CA) according to the manufacturer's instructions. RNA concentration and purity was determined with the NanoDrop 2000 Spectrophotometer (Thermo Scientific, Wilmington, DE) and agarose gel electrophoresis was used to assess RNA integrity. The A_260_/A_280_ ratio was 1.94-2.2. for all samples. The RNA integrity numbers (RIN) were 9.5-9.8 as determined by RNA 6000 Nano kit in an Agilent 2100 Bionalyzer (Agilent technologies, Santa Clara, CA).

### Validation of mRNAs and lncRNAs by quantitative real-time PCR (qRT-PCR)

Total RNA reverse transcription was conducted using PrimeScript^™^ RT reagent kit (TaKaRa) according to the manufacturer's instructions with either the oligo (dT) primers or specific RT primers. cDNA was quantitated by real-time PCR using the primers listed in Table 4 (Integrated DNA Technologies, Coralville, IA). Each sample was run in triplicate in 20μl reactions with 0.4 μM forward and reverse PCR primers and 10μl of Advanced Universal SYBR Green Supermix (Bio-Rad Laboratories, Hercules, CA) in a Bio-Rad CFX96 real-time PCR system. The PCR cycle parameters were set as follows: initial denaturation at 95°C for 1 min followed by 40 cycles of 95°C for 15s, 60°C for 15s, and 72°C for 45s. Relative expression was determined by normalization to GAPDH using the 2^−ΔΔCt^ method. The experiments were performed in triplicate.

**Table 4:** Primers for RT-qPCR

Primer names	Sequences
TCONS_00034035	F:TTCCAGAGGTCCTGAGTTCAA R:CACCAGAAGAGGGCATCG
TCONS_00034035	F:CCCAGTCTCCATCCTCCA R:CAGACCTACTGCCACTGAACC
TCONS_00034216	F:CCACTCAGGCAGAGGTCAAA R:CTGAGGACTGAAACACCAGCA
TCONS_00033710	F:TCATTGCGCCAATTAGGGA R:CGTCCTGCTTGACCTTTCCTT
TCONS_00058568	F:AGATCCTCCATGAAATGCTTCC R:AAGTTTCCAACTCCAGCCAAG
TCONS_00042175	F:AAGAGGCATCGTAGCGTGGAC R:GAAGGACGGGCTGTGAGTGT
TCONS_00018621	F:AAGGAAAGAATGTTTTGTGGAC R:TTTGGTCAAGTTGTTTCCATTA
TCONS_00047728	F:GGGATGCAGTGGGGTGAC R:TTCTTTGTTGATACAGGGAGGC
TCONS_00070166	F:ATGAGAAACAGGGGTATGCTAAAG R:GGTGAGTGGCTGTCCCAAAG
TCONS_00033040	F:TTTCAGATGCTCCAGTTGTTGTG R:CTTCTGGGGAATGTGGAGTAAGT
RatNP-3b	F:CGCCAAAGTCTGAAACCACA R:CCCTCCAAAGAATACGGAAATA
S100a9	F:AGCGCAGCATAAGCACCA R:GATCAACTTTCCCATCAGCATC
Smoc2	F:AGATATTGCCTCACGCTACCC R:TGATCACAGGAGGATGCTGAA
P2×7R	F:CTTCGGCGTGCGTTTTG R:AGGACAGGGTGGATCCAATG
Slc47a1	F:GACATGGCTTGTCTTCTGCTTG R:GCAGGATAACCCCTACGTGCTTT
GAPDH	F:GAAAGCCTGCCGGTGACTAA R:AGGAAAAGCATCACCCGGAG

RT-qPCR: reverse transcriptase quantitative polymerase chain reaction;

F: Forward; R: Reverse.

### Western blotting

Total protein extract was prepared from T8-12 spinal cord tissues with protein lysis buffer. Western blotting was performed as described previously [65, 66]. 50μg of protein samples were separated by SDS-PAGE and transferred onto PVDF membranes (Millipore, USA). Then, the PVDF membrane was blocked with 5% skimmed milk in TBST for 1h at room temperature. Then, the membranes were incubated with the following primary antibodies: P2×7R (Boster, China), S100A9 (Boster, China), Akt (Cell Signaling Technology, USA), P-Akt (Cell Signaling Technology, USA), Bcl-2 (Affinity Biosciences, USA), Bax (Affinity Biosciences, USA), and Caspase-3 (ABclonal, USA). Further, after washes with 1X TBST, the membrane was incubated with anti-mouse or goat HRP-conjugated secondary antibodies (Abbkine, USA). This was followed by protein detection by ECL Assay Kit (Bipec Biopharma). β-actin (Abbkine, USA) was used as internal control. Band intensity was quantified using ImageJ software (1.44 P) and the expression of target proteins relative to β-actin control was determined.

### Gene ontology (GO) and KEGG pathway analysis

The mRNA expression profiles from control and I/R group were screened by volcano plot filtering and differential mRNA expression was determined with Gene Ontology program (http://www.geneontology.org), which classified the data into biological processes, cellular components and molecular functions. Moreover, the key regulatory pathways in the spinal cord responding to I/R-induced AKI were analyzed using the Kyoto Encyclopedia of Genes and Genomes (KEGG) pathway analysis (http://www.genome.jp/kegg).

### Statistical analysis

Data were presented as mean±SEM. Statistical analysis was performed using a t test between two cohorts. Data processing and subsequent quantitative normalization were performed using GeneSpring GX v12.1 software (Agilent Technologies). Differentially expressed mRNAs and lncRNAs between control group and I/R group were identified through fold change filtering. P value <0.05 was considered statistically significant. Hierarchical clustering and combined analysis was performed with homemade scripts (Multi-Experiment Viewer clustering).

## SUPPLEMENTARY MATERIALS FIGURES AND TABLE



## Abbreviations

I/R: ischemia/reperfusion
AKI: acute kidney injury
CPC: coding potential calculator
CNCI: coding-non-coding index
GO: gene ontology
KEGG: Kyoto Encyclopedia of Genes and Genomes
SG: stellate ganglia
SCG: superior cervical ganglia
SD: Sprague-Dawley
PAS: periodic acid-Schiff reagent
SCr: serum creatinine
qRT-PCR: quantitative real-time PCR
RIN: RNA integrity numbers.
