# Supplementary material for: Differential gene and lncRNA expression in the lower thoracic spinal cord following ischemia/reperfusion-induced acute kidney injury in rats
Source: Oncotarget. 2017 Jun 20;8(32):53465–81. doi: 10.18632/oncotarget.18584 (PMC5581123; doi:10.18632/oncotarget.18584)
Supplement: Supplementary file 1 [file oncotarget-08-53465-s001.pdf]

Differential gene and lncRNA expression in the lower thoracic spinal cord following ischemia/reperfusion-induced acute kidney injury in rats

SUPPLEMENTARY MATERIALS

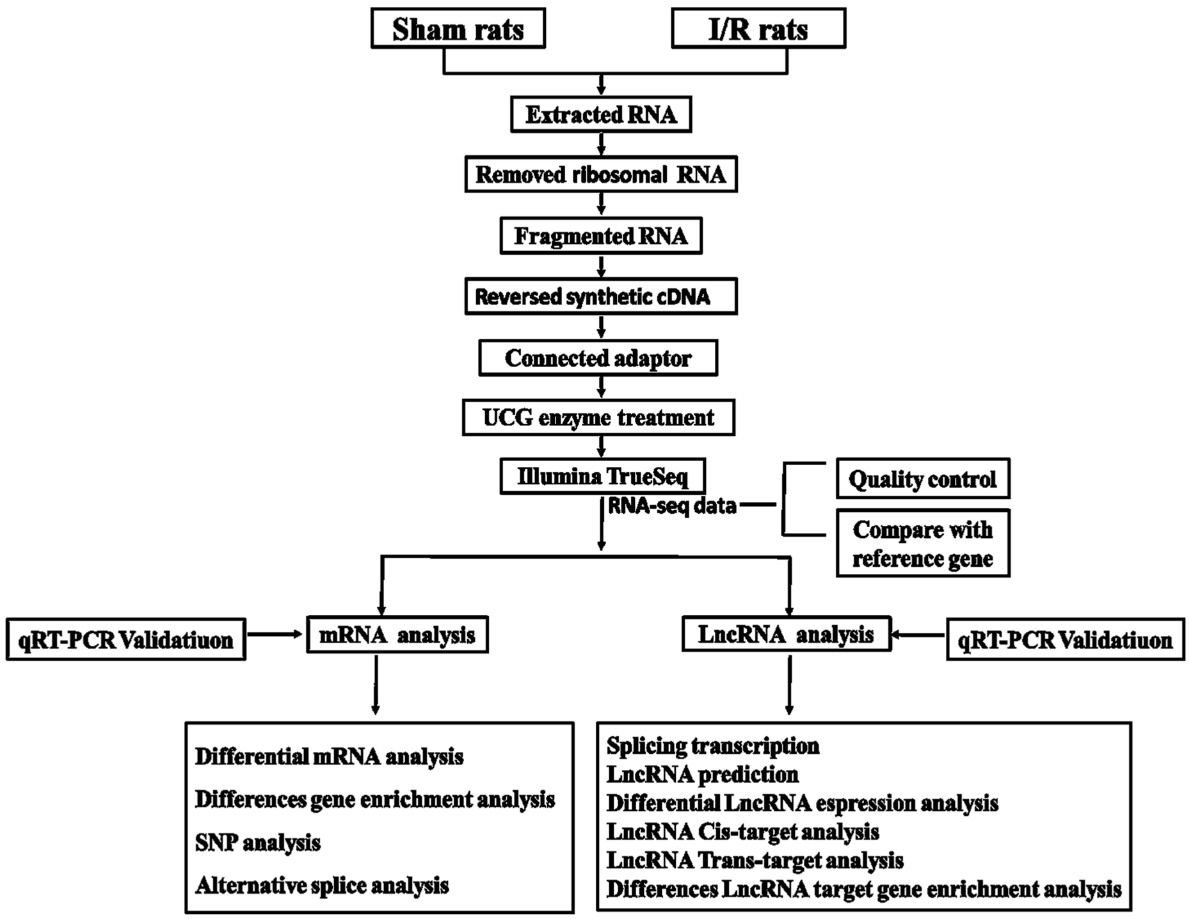

Supplementary Figure 1: The sequencing operation flow chart of the experimental process and the multi-step strategy used in this study.

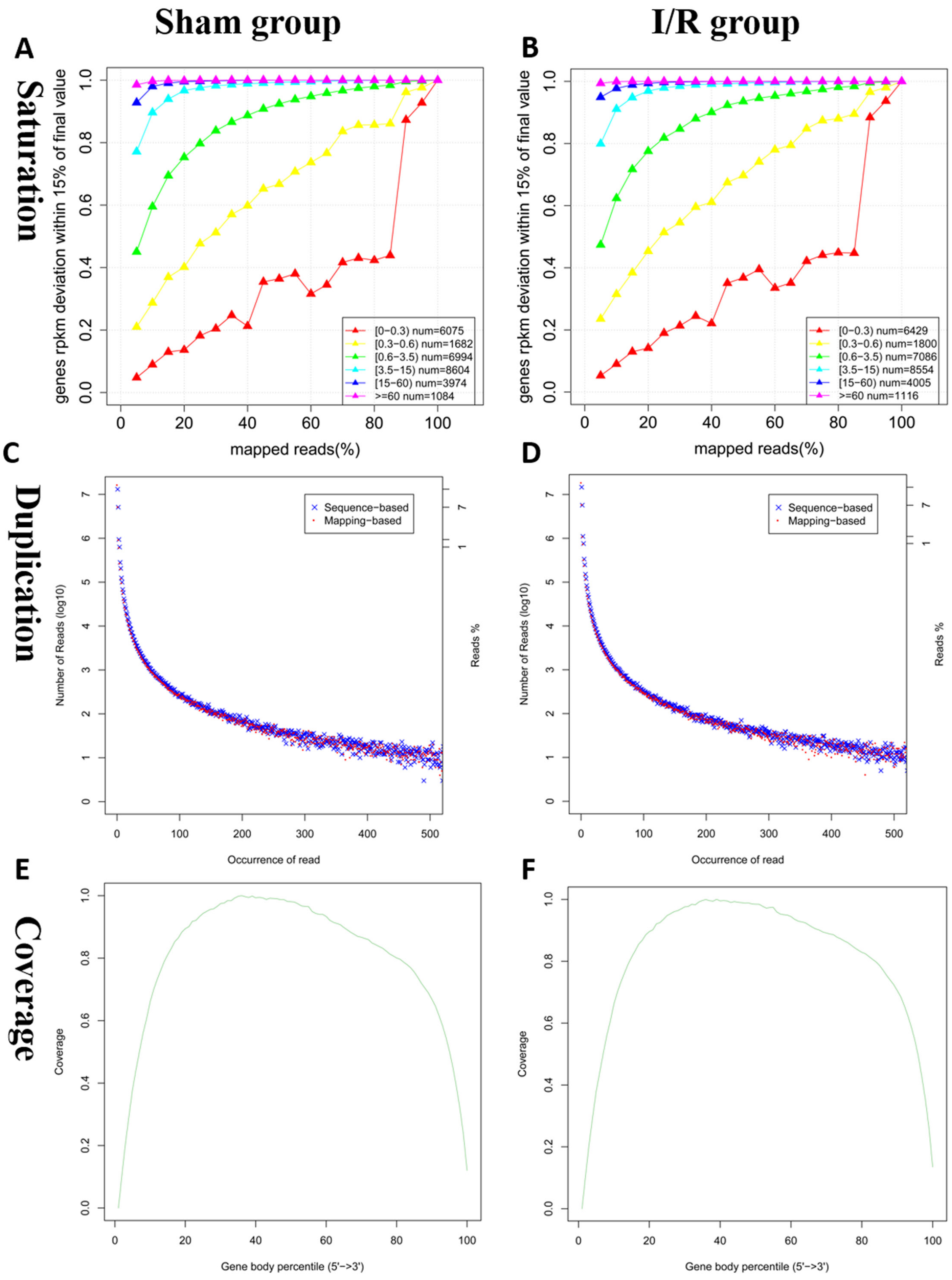

Supplementary Figure 2: Transcriptome quality control. Perform quality assessment of overall transcriptome from the saturation, duplication reads and coverage. **(A and B)** Saturation analysis. **(C and D)** Duplication reads analysis. **(E and F)** Coverage analysis.

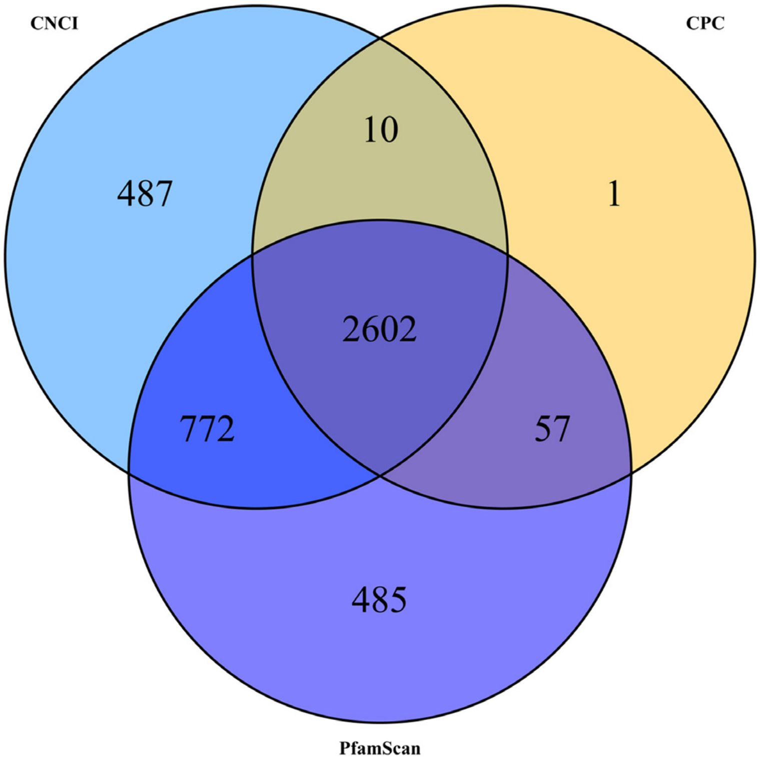

Supplementary Figure 3: Venn diagram showing the number of novel LncRNA. The novel predicted LncRNAs were obtained by filtering using the CPC (Coding Potential Calculator) analysis, CNCI (Coding-Non-Coding Index) analysis, Pfam protein structure domain analysis, and then overlap analysis the filtered predicted LncRNAs

Supplementary Table 1: Comparison of Clean Data and reference genome

Type	Sham1 group	Sham2 group	Sham3 group	I/R1 group	I/R2 group	I/R3 group
**Total reads**	86787136	86890544	90546378	92926314	89944647	88648926
**Total mapped**	73534839 (84.73%)	78045087 (89.82%)	81328757 (89.82%)	85002705 (91.47%)	78737544 (87.54%)	77071376 (86.94%)
**Multiple mapped**	4599718 (5.30%)	4848492 (5.58%)	4952887 (5.47%)	5450846 (5.87%)	5063884 (5.63%)	4849096 (5.47%)
**Uniquely mapped**	68935121 (79.43%)	70659390 (81.32%)	74755090 (82.56%)	79551859 (85.61%)	73556732 (81.78%)	71380115 (80.52%)
**Left mapped**	37118499 (42.77%)	39430929 (45.38%)	41778099 (46.14%)	42945236 (46.21%)	39746540 (44.19%)	38358390 (43.27%)
**Right mapped**	36416340 (41.96%)	38023302 (43.76%)	40102991 (44.29%)	42057469 (45.26%)	38658209 (42.98%)	37507361 (42.31%)
